# Retrospective evaluation of early thrombosis in transjugular intrahepatic portosystemic polytetrafluoroethylene-coated shunts under 2-day postinterventional heparinization

**DOI:** 10.1038/s41598-022-14388-3

**Published:** 2022-06-22

**Authors:** Holger Goessmann, Verna Schuffenhauer, Arne Kandulski, Kilian Weigand, Ernst-Michael Jung, Wibke Uller, Gregor Scharf, Cristian Stroszczynski, Niklas Verloh

**Affiliations:** 1 Department of Diagnostic and Interventional Radiology, University of Leipzig Medical Center, Leipzig, Germany; 2 Department of Radiology, University Hospital Regensburg, 93042 Regensburg, Germany; 3 Department of Internal Medicine I, Gastroenterology, Endocrinology, Rheumatology, and Infectious Diseases, Regensburg University Hospital, Regensburg, Germany; 4 Department of Diagnostic and Interventional Radiology, Faculty of Medicine, Medical Center University of Freiburg, University of Freiburg, Hugstetter Straße 55, 79106 Freiburg, Germany

**Keywords:** Gastroenterology, Liver cirrhosis, Drug regulation

## Abstract

The development of acute thrombosis within the TIPS tract may be prevented by prophylactic anticoagulation; however, there is no evidence of the correct anticoagulation regimen after TIPS placement. The purpose of this single-center retrospective study was to evaluate the short-term occlusion rate of transjugular intrahepatic portosystemic shunts (TIPSs) with polytetrafluorethylene (PTFE)-coated stents under consequent periprocedural full heparinization (target partial thromboplastin time [PTT]: 60–80 s). We analyzed TIPS placements that were followed up over a six-month period by Doppler ultrasound in 94 patients and compared the study group of 54 patients who received intravenous periprocedural full heparinization (target PTT: 60–80 s) without any other anticoagulation to patients with prolonged anticoagulation medication. The primary endpoint was TIPS patency after six months. The primary patency rate was 88.3% overall, and in the study group, 90.7%, with an early thrombosis rate of 3.2% (study group: 1.9%) and a primary assisted patency rate of 95.7% (study group: 96.3%). In the study group, one case of TIPS thrombosis occurred on the 23rd day after TIPS placement. Two patients underwent reintervention because of stenosis or buckling. Moreover, the target PTT was not attained in 8 of the 54 patent TIPSs. Four patients had an increased portosystemic pressure gradient, without stenosis, and the flow rate was corrected by increasing the TIPS diameter by dilation. Two-day heparinization seems sufficient to avoid early TIPS thrombosis over a six-month period.

## Introduction

Transjugular intrahepatic portosystemic shunts (TIPSs) are now routinely used to manage portal hypertension complications^1–4^. They are indicated for the treatment of refractory ascites, to prevent variceal rebleeding, or to prevent massive variceal bleeding. Experience with this treatment has existed since 1989^5^ and has been continuously improved technically since then. TIPS is a nonoperative intervention to divert portal flow into the body's circulation, thereby relieving the portal vein and collateral circulation.

Instead of bare-metal stents, polytetrafluorethylene (PTFE)-coated stents are routinely used. Bare metal stents do not prevent neointimal proliferation, which gradually reduces their inner diameter, affecting TIPS outcomes for the usual indications. Stents coated with PTFE were developed to improve the long-term patency of TIPSs. The first retrospective studies confirmed that these stents have a lower percentage of thrombosis^6–11^. These findings were supported by a randomized study in which PTFE-coated stents showed superiority in long-term patency^12,13^ and were controlled in a multicenter, randomized, single-blinded (with blinding of patients only) parallel-group trial. The latter study observed a significant 39% reduction in dysfunction for coated stents compared to bare-metal stents^14^.

The CIRSE standard of practice guidelines recommends post-interventional anticoagulation only in cases with Buddy-Chiari syndrome^15^. On the other hand, the S2k guideline of the German Society of Gastroenterology, Digestive and Metabolic Diseases (DGVS) "Komplikationen der Leberzirrhose" ("Complications of Liver Cirrhosis") states that no recommendation can be made for optimal anticoagulation after TIPS implantation due to a lack of study data and that the current lack of clarity about the correct anticoagulation regimen after TIPS implantation or whether anticoagulation is still necessary at all should be the subject of future studies^16^. Steib et al*.*^17^ 2019 conducted a survey study on the current procedure at 43 German hospitals, which showed an enormous postinterventional variation in antithrombotic measures.

Considering the different possible anticoagulation regimens, the purpose of this retrospective study was to investigate whether periprocedural complete intravenous heparinization for two days monitored by partial thromboplastin time (PTT) is sufficient to avoid early thrombosis of a newly placed TIPS using stent grafts (Viatorr®, Gore Medical).

## Results

Across the 94 included patients, 27 were female (28.7%), and 67 were male (71.3%). The patients ranged in age from 19 to 80 years at the time of TIPS implantation, with a mean age of 57.6 ± 10.9 years (Table 1). The most frequent indication for TIPS placement was existing refractory ascites (n = 64), including 16 Patients with a combination of refractory ascites and gastrointestinal varices. In total, there were 40 patients with gastrointestinal varices, with esophageal varices accounting for n = 20, GI varices accounting for n = 11, and combined varices accounting for n = 9. In 68 procedures, a shunt was established between the right hepatic vein and the right portal vein (Table 1). Establishment of a shunt from the right hepatic vein to the central portal vein was performed in four cases and to the left portal vein branch in three cases. In 17 implantations, a shunt was established starting from the middle hepatic vein to the right portal vein (n = 12), to the left portal vein (n = 4), and once to the central portal vein. Two patients received implantation from the left hepatic vein: one to the left portal vein and one to the central portal vein. The indication for TIPS implantation as well as the initial position were not statistically significantly related to the primary patency rate or the occlusion rate at six months.

**Table 1:** Patient characteristics.

	All (n = 94)	Study group (n = 54)	Add-on anticoagulation (n = 40)	p-value
Age, years	57.6 ± 10.9	58.2 ± 9.8	56.9 ± 12.3	n.s
Sex (male), n (%)	67 (71.3)	35 (64.8)	32 (80.0)	n.s
Indication *				n.s
Therapy refractory ascites	64	35	29	
Gastrointestinal varices	40	18	22	
Acute portal vein thrombosis (No flow on the main branch)	4	0	4	
Others, e.g., Budd Chiari syndrome	6	1	5	
TIPS location				n.s
Right hepatic vein to portal vein, n	75	44	31	
Middle hepatic vein to portal vein, n	17	10	7	
Left hepatic vein to portal vein, n	2	0	2	
Preinterventional laboratory results				
Total bilirubin (mg/dl)	1.5 ± 1.1	1.6 ± 1.2	1.3 ± 0.8	n.s
Serum creatinine (mg/dl)	1.2 ± 1.0	1.4 ± 1.3	1.0 ± 0.4	0.022
Prothrombin time (s)	40.5 ± 8.1	41.1 ± 8.6	39.6 ± 7.3	n.s
INR	1.3 ± 0.2	1.4 ± 0.3	1.3 ± 0.2	n.s
Model for end-stage liver disease (MELD) score	13.7 ± 4.3	13.8 ± 4.6	11.3 ± 3.4	n.s
Preinterventional pressure gradient (mmHg)	15.3 ± 5.1	15.7 ± 5.1	14.7 ± 5.1	n.s
Postinterventional pressure gradient (mmHg)	6.9 ± 2.6	6.9 ± 2.1	7.1 ± 3.1	n.s

* more than one possible; n.s., not significant.

The characteristics of the study population are shown in Table 1.

Before TIPS insertion, the pressure gradient was 15.3 ± 5.1 mmHg (study group: 15.7 ± 5.1 mmHg), and after insertion 6.9 ± 2.6 mmHg (study group: 6.9 ± 2.1 mmHg). TIPS placement achieved a mean reduction in portal-system pressure by 8.6 ± 4.1 mmHg (study group: 9.3 ± 4.1 mmHg).

All patients received postinterventional anticoagulation for at least two days with unfractionated heparin administered via an injector and a loading dose on the day of the intervention. Twenty patients had at least one preexisting anticoagulant therapy before the TIPS procedure (Table 2), and post-interventional anticoagulation was prolonged in 40 patients (20 patients without preexisting medication). The 54 patients who received postinterventional unfractionated heparin solely and without prolonged anticoagulation (study group) were investigated further and compared with those who received prolonged or additional anticoagulation (add-on anticoagulation).

**Table 2:** Anticoagulation scheme.

Preexisting medication	Intervention	Prolonged anticoagulation after day 3
NONE (n = 74)	Unfractionated heparin for 3 days	None (n = 54*)
		Fractionated heparin (n = 14)
		Prolonged unfractionated heparin (n = 2)
		Thrombocyte aggregation inhibitor (n = 2)
		Direct Xa inhibitor (n = 1)
		Thrombocyte aggregation inhibitor + fractionated heparin (n = 1)
Fractionated heparin (n = 9)	Unfractionated heparin for 3 days	Fractionated heparin (n = 7)
		Prolonged unfractionated heparin (n = 2)
		Thrombocyte aggregation inhibitor + fractionated heparin (n = 1)
Thrombocyte aggregation inhibitor (n = 9)	+ unfractionated heparin for 3 days	Thrombocyte aggregation inhibitor (n = 7)
		+ fractionated heparin (n = 2)
Thrombocyte aggregation inhibitor + fractionated heparin (n = 1)	Thrombocyte aggregation inhibitor + unfractionated heparin for 3 days	Thrombocyte aggregation inhibitor + fractionated heparin (n = 1)
Unfractionated heparin (n = 1)	Unfractionated heparin for 3 days	Fractionated heparin (n = 1)

*Study group.

For further analysis, the study group was investigated to reach the target partial thromboplastin time. The pre-interventional prothrombin time was 40.5 ± 8.1 s (min, 28.8; max, 60.4 s) for the study group, and the target PTT was reached in 46 cases (85.2%) within three days post-interventionally. No statistically significant association was found between achievement of the target PTT and development of mechanically or thrombotic-related TIPS dysfunction.

All TIPS procedures showed no sign of in-stent thrombosis on imminent postinterventional ultrasound (median of 3.5 days after TIPS placement), with a mean flow rate of 124 ± 36 m^3^/s, as measured with Doppler sonography. Within the follow-up of 6 Months, a flow velocity below 50 cm/s was observed in 9 cases (4 within the study group), and in 11 cases (8 within the study group), the flow rate within the TIPS tract reached values greater than 200 cm/s. Of these 20 patients with abnormalities on sonography (12 in the study group), reintervention was performed only in 3 patients (2 in the study group) due to coexisting clinical signs of TIPS dysfunction. No correlation was found between the rates of reintervention and the findings by CCDS. The referring colleagues primarily relied on clinical signs of TIPS dysfunction rather than changes found on sonography alone.

Fifteen patients had clinical signs of a stent dysfunction without any abnormalities on sonography, leading to an invasive TIPS control through a transjugular route. Clinical signs of TIPS dysfunction were refractory ascites, indicating a reduced TIPS patency, or hepatic encephalopathy, indicating an increased flow throw the TIPS tract.

**Figure 1:**
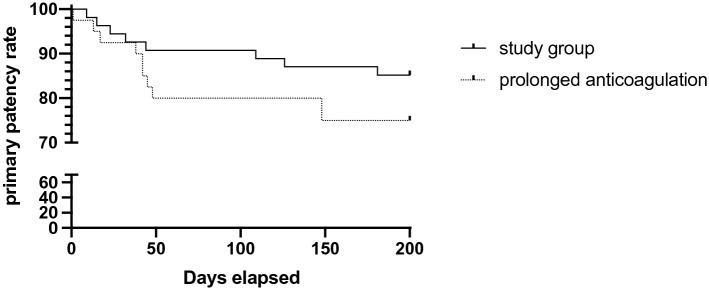
primary patency rate. Kaplan–Meier curve compares the primary patency rate between patients with unfractionated heparin for three days (study group) and patients with prolonged anticoagulation.

Overall, a reintervention was performed within the follow-up period of 6 months in 18 cases, with 8 cases in the study group due to clinical signs of TIPS dysfunction. The primary patency rate (Table 3) was 88.3% (study group: 90.7%), with an early thrombosis rate of 3.2% (study group: 1.9%), and the primary assisted patency rate was 95.7% (study group: 96.3%). There was no significant difference between the study group and patients with prolonged anticoagulation in the patency rate and the time points of the events (Fig. 1).

**Table 3:** Shunt patency at 6 months after TIPS placement.

	All	Study group	Add-on anticoagulation
Ultrasound abnormalities, n	20/94	12/54	8/40
Flow velocity < 50 cm/s	9/20	4/12	5/8
Flow velocity > 200 cm/s	11/20	8/12	3/8
Increased portal pressure, n^+^	4/94	2/54	2/40
TIPS dysfunction, n	11/94	5/54	6/40
Thrombosis*	3/11	1/5	2/6
Stenosis‡	6/11	3/5	3/6
Buckling#	2/11	1/5	1/6
Diameter reduction, n	3/94	1/54	2/40

^+^Increased portal-system pressure gradient with no sign of stenosis, corrected by increasing the diameter of the TIPS by dilation.

*Treated with recanalization and dilatation of the TIPS tract.

^‡^Stenosis, because of intimal hyperplasia, corrected by dilation.

^#^Corrected by the placement of an additional stent.

In the study group, six patients had refractory ascites (mean: 67.8 ± 69.7 days; median: 38 days). All six patients received invasive control by catheterization: one case of buckling, corrected by the placement of an additional stent, and one case of stenosis, corrected by dilation, were found. The other two patients in the study group had an increased portosystemic pressure gradient, without stenosis, measured by catheterization, and the flow rate was corrected by increasing the diameter of the TIPS by dilation. Development or exacerbation of hepatic encephalopathy occurred in one patient (109 days after TIPS placement) and was treated by TIPS diameter reduction.

**Figure 2:**
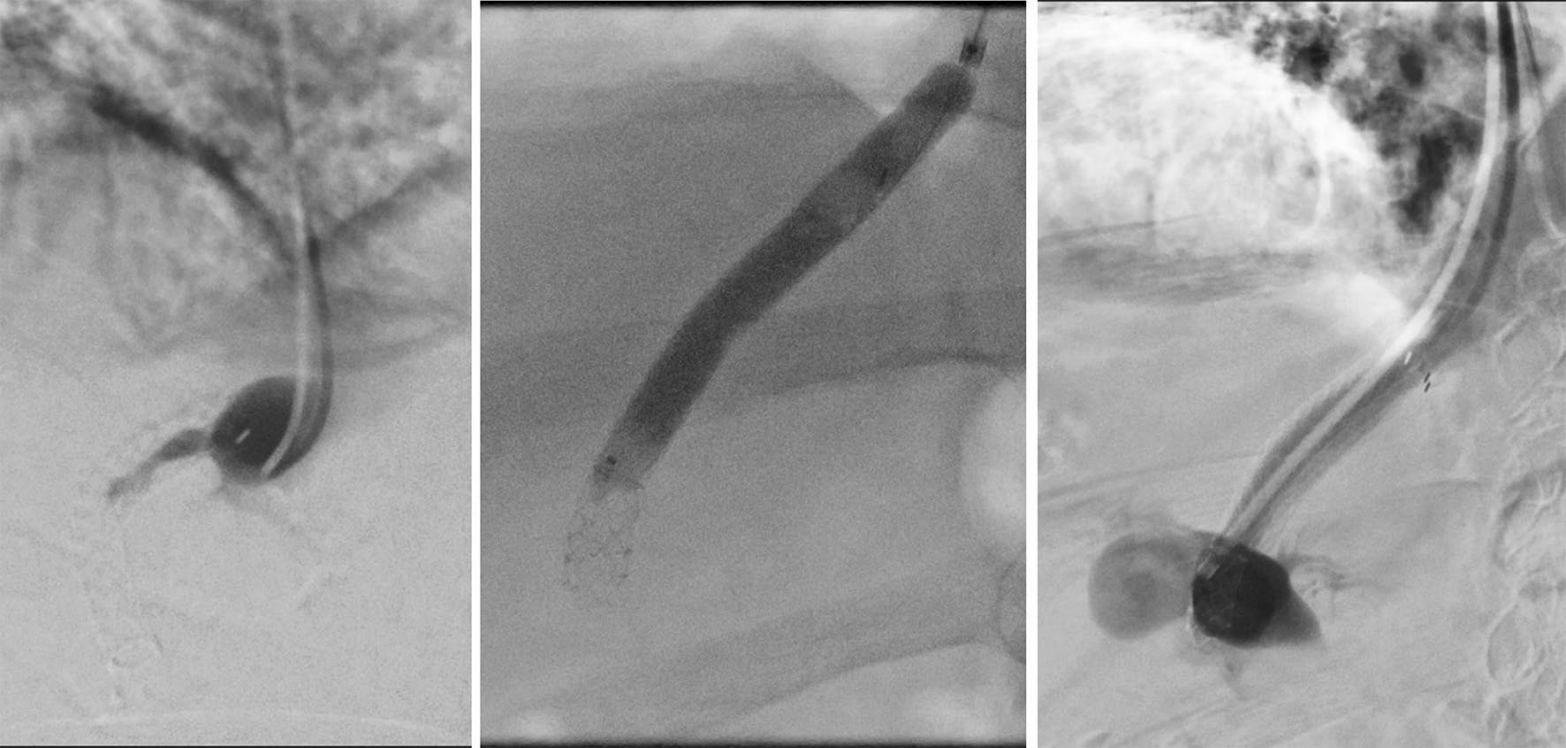
TIPS occlusion. Angiographic evidence of TIPS occlusion at day 23 postinterventional. The patient was previously readmitted with hydropic decompensation and sonographic evidence of early TIPS occlusion. The patency of the TIPS tract was successfully restored by balloon PTA.

**Figure 3:**
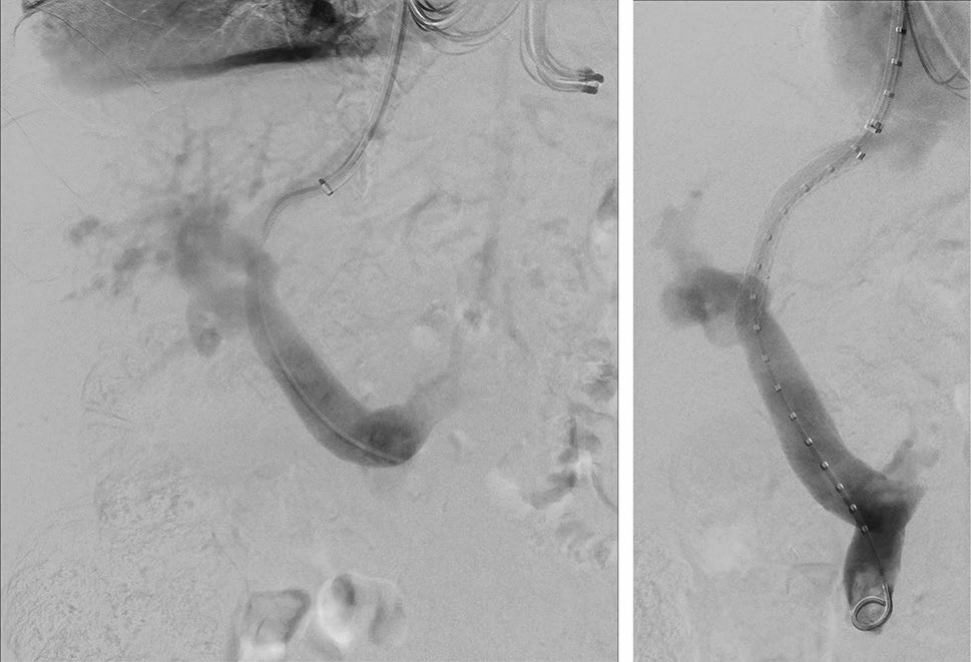
TIPS insertion. The initial TIPS in a 79-year-old male patient was placed from the middle hepatic vein to the left portal vein due to refractory ascites with esophageal varices (Gore Viatorr stent; 8 mm diameter; 60 × 20 mm).

**Figure 4:**
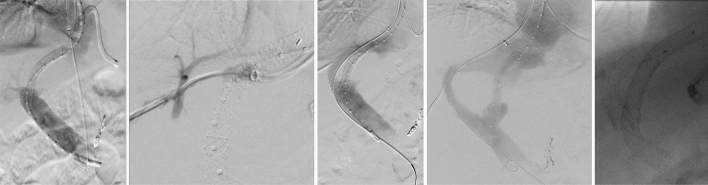
Second TIPS placement. A second TIPS implantation from the right hepatic vein to the right portal vein was necessary in the same patient due to persistent gastrointestinal hemorrhage. The initial TIPS remained patent.

Occlusion occurred in one patient with primary biliary cholangitis and liver cirrhosis on day 23 after TIPS implantation (Fig. 2). The patient received a Gore Viatorr stent (8 mm, 60 × 20 mm) implanted between the middle hepatic vein on the left portal vein branch due to refractory ascites with esophageal varices at the age of 79 years (Fig. 3). No antithrombotic therapy was given preoperatively, and the patient received anticoagulation therapy according to the standardized protocol. A total of 4 reinterventions were performed during follow-up before a second TIPS implantation on postoperative day 100. On day 100, a second TIPS was implanted due to persistent bleeding and dysfunction of the first (Fig. 4).

Six patients had a reintervention in the group with prolonged anticoagulation due to a TIPS dysfunction. Two cases of in-stent thrombosis, one due to preexisting portal vein thrombosis, aided with dilatation, and in case of the preexisting portal vein thrombosis, reinstalling the direct lysis catheter. One case of buckling, corrected by the placement of an additional stent, and three cases of stenosis, corrected by dilation, were found. Four had an increased portosystemic pressure gradient, without stenosis, measured by catheterization, and the flow rate was corrected by increasing the diameter of the TIPS by dilation.

## Discussion

Historically, one of the main adverse events of the TIPS procedure was the average dysfunction rate of 50% after one year^18,19^. With a 1-year primary patency rate of 81%—84%, the development of ePTFE-coated stents marked a new era of percutaneous portal decompression^20,21^. In several studies^9,20–23^, the ePTFE-coated Viatorr stent graft was demonstrated to be superior to conventional stents, not only because of primary patency rates within the first year but also for lower rates of recurrent bleeding and mortality^20^. Close monitoring of TIPSs in combination with prophylactic intervention has resulted in a decreased incidence of symptom recurrence and a lowering of shunt revision rates; some studies with the Viatorr stent graft even suggest that the frequency of routine TIPS monitoring can be reduced due to the superior patency rates of the ePTFE-coated Viatorr stent graft^24–26^. For the short term (up to six months after placement), data indicate that coated stent grafts can significantly reduce the risk of dysfunction^10,12,13,27–29^. As the dysfunction rate was relatively low in this study, our study supports the fact that dysfunctions in coated stent grafts are generally low.

The development of acute thrombosis within the TIPS tract may be prevented by prophylactic anticoagulation; however, there is uncertainty about the correct anticoagulation regimen after TIPS or whether anticoagulation is still necessary after stent-graft implantation^16^. The Vienna TIPS Study Group proposed a patient management and documentation strategy before and after TIPS implantation^30^. Independent of the implanted TIPS, anticoagulation with low-molecular-weight heparin was administered for three days, followed by clopidogrel (75 mg/day) for six months. In a randomized study, Sauer et al*.*^31^ investigated the shunt patency of the Palmaz-TIPS-Stent, a bare-metal stent, in prolonged anticoagulation therapy after three days of heparinization with phenprocoumon: They found that the patency of the Palmaz-TIPS-Stent is increased in prolonged anticoagulative therapy with phenprocoumon for three months (no occlusion in the study group, compared to five complete occlusions in the control group) but that further anticoagulative treatment seems unnecessary^31^. In a prospective, noninterventional study (TIPS Registry) in 10 centers, Bettinger et al*.*^32,33^ compared 412 patients who received TIPS placement between 2006 and 2011 with either a Viatorr stent (n = 227) or an uncoated stent (n = 185). They found that patients who received an uncoated stent benefited from peri-interventional anticoagulation (low-molecular-weight heparin and ASS). In contrast, patients who received a coated Viatorr stent did not appear to benefit from anticoagulation^33^. The Viatorr group had a lower need for revision in the first year, but the two groups had a balanced rate of revisions (30% in both groups) after two years^32^.

The present study evaluated the success rate of the standard postinterventional anticoagulant regimen with periprocedural full heparinization (target PTT: 60–80 s) for two days. The primary patency rate of all TIPS devices in the present study was 88.3% (study group: 90.7%), with an early thrombosis rate of 3.2% (study group: 1.9%). The results of our study are in line with reported thrombosis rates of less than 5% in the literature^34,35^. The most common cause of these occlusions is thrombus formation due to obstruction of the blood flow by poorly placed stents^34^ or stent migration^34,35^. It is known that the cause of shunt dysfunction is often attributed to suboptimal, incomplete coverage of the tract, which can cause TIPS displacement or kinking and promote the development of thrombosis^35^. In addition, the geometry of the TIPS track itself, and the angle of portal venous inflow, also influence the patency rate^36^. This study suggests that in patients with correct stent positioning, two days of heparinization with a target PTT of 60–80 s is sufficient to prevent early thrombosis. However, on the other hand, prolonged anticoagulation cannot prevent thrombosis in mechanically induced TIPS dysfunction. Due to the widely varying definitions of TIPS dysfunction in the literature, a direct comparison regarding patency rates and dysfunction to other work is limited. However, with a patency of 88.3% or 90.7% (study group), the present investigation shows comparable patency rates to those in studies with PTFE-coated stents ranging between 87.4% and 92.7%^20,35,37^ (Table 4).

**Table 4:** Comparative studies.

	n	Anticoagulant regimen	Primary patency rate	Thrombosis rate
Vignali et al*.*^35^	113 (#)	No peri- and postinterventional anticoagulation	91.9%	n/a
Kijak^38^	14	Low-molecular-weight heparin for 14 days	92.8%	0%
Entzian^37^	50	periinterventional heparin injection of 5000 I.U. (n = 44)	92.7%	4%
Jahangiri et al*.*^56^	174	n/a	94.1% (1 year)	9.8% (1 year)
Perello et al*.*^39^	132	No peri- and postinterventional anticoagulation	77 ± 7.4% (1 year)	n/a
Present study	54	Periprocedural full heparinization (target PTT 60–80 s)	90.7%	1.9% (n = 1)

The Viator stent was used in each of the studies reported, if not otherwise specified. The primary patency and thrombosis rates are given for six months, if not otherwise specified.

(#) Modification of TIPSs at primary installation with one additional coated stent each (n = 5).

Comparing our results to different anticoagulation schemes in Viatorr stents revealed similar patency rates. Kijak et al*.*^38^ used an anticoagulant regimen with postinterventional administration of subcutaneously administered low-molecular-weight heparin (20 mg enoxaparin sodium) for 14 days without a peri-implant heparin dose and reported primary and secondary patency rates of 92.8% and 100%, respectively, at six months with only one case of venous non thrombotic stenosis proximal to the stent (7.2%) at three months and no TIPS thrombosis. A primary patency rate of 88% was reported by Entzian et al*.*^37^ with an anticoagulation scheme of only a simple periinterventional heparin injection of 5000 I.U. with no additional postinterventional anticoagulant therapy. Among other reasons, reintervention resulted from partial Viatorrs® stent thrombosis in two cases (6.5% of patients with a follow-up period up to 6 months) at 5 and 109 days^37^. Comparatively, it can be assumed that patients might benefit from three days of full heparinization following a periinterventional heparin injection of 5000 I.U. (1.9% in-stent thrombosis).

In a long-term follow-up study of Viatorr® stents without anticoagulation during or after TIPS placement, shunt dysfunction occurred in 36% of patients over 12 years, resulting in reintervention in 29% of the cases^39^. Of these, 74% of reinterventions occurred within the first two postinterventional years, and TIPS occlusion occurred in 12% of the cases^39^. At one year, the calculated primary patency rate was 77 ± 7.4%, which was significantly lower than the primary patency rate of studies with peri- and postinterventional anticoagulant regimens. Analyzing the Kaplan–Meier curve of Perello et al*.*^39^ revealed that in their study, most TIPS dysfunctions took place directly post-interventionally or within the first six months, which could be due to early complications with TIPS dysfunctions due to a lack of anticoagulation.

The liver is the central organ in the synthesis of plasmatic coagulation factors. In the case of chronic diseases of the liver, paradoxical procoagulatory conditions often occur in addition to bleeding complications^40–42^. There are no guidelines or large prospective studies on anticoagulation therapy in cirrhosis patients for the treatment or prevention of venous thromboembolism. The prevention or treatment of thrombosis in cirrhosis patients often results from extrapolating therapy decisions in noncirrhotic patients^43–46^. Advanced liver insufficiency and liver failure are potential contraindications for anticoagulation with a vitamin K antagonist (VKA) or non-vitamin K antagonist oral anticoagulant (NOAK)^41^. Together with antithrombin, a natural protein in the body, unfractionated heparin (UFH) is a fast-acting blood thinner that blocks the development of clots: UFH binds to antithrombin and amplifies its potential to inhibit two clotting factors: Factor Xa and Factor IIa, thus acting quickly to prevent clot formation. UFH decreases rapidly when the infusion is stopped or reversed using the antidote protamine^47^. These pharmacokinetic properties allow a demand-oriented and manageable anticoagulative therapy in patients with liver cirrhosis.

Patients with LC have a complex coagulation status due to global hemostatic changes affecting both pro- and anticoagulant factors. Although LC significantly affects the production and regulation of these factors, the rebalancing of factors in compensated patients results in overall normal coagulation, a so-called "rebalanced hemostasis "^48,49^. Our results also indicated that patients' limited coagulation situation might make seeking postinterventional anticoagulative therapy PTT targets unnecessary. In 14.8% of the study group, target PTT was not achieved on the third postinterventional day and was discontinued according to the given standard. The absence of in-stent thrombosis in this small subgroup indicates that the target PTT achievement might not be necessary; further (prospective) studies are required to confirm this.

Due to this study's retrospective character, there are a few limitations. As the data collection showed, clinicians were not strictly bound to the anticoagulation protocol. Other anticoagulant therapies were maintained due to preexisting comorbidities, or anticoagulation was continued for an extended period, depending on the clinical situation. In addition, no patients were included without post-interventional anticoagulation; Therefore, no statement can be made as to whether a two-day heparinization can prevent thrombosis compared to no anticoagulation. The control group in this study was very heterogeneously with different anticoagulation regimes, thus limiting the final statement for this study cohort.

Comparing our study group results to the literature findings is extremely difficult or even impossible due to the diverse study designs, different definitions, and, in some cases, fuzzy or missing descriptions of the parameters. On the one hand, various parameters are used to assess TIPS dysfunction, and on the other hand, the criteria for TIPS dysfunction are not precisely defined. For example, sonographic findings or invasive pressure gradient measurements are used. There are no consensus criteria in the literature concerning TIPS patency; they differ in the studies, sometimes significantly, and are also formulated imprecisely in some cases.

An additional obstacle to classifying the results obtained from the present investigation in the comparative literature is the lack of studies focusing on postinterventional anticoagulant therapy in TIPS implantation, especially concerning the early thrombotic closure rate. Table 2 shows many different approaches in use, even in this single-center, to prevent post-interventional thrombosis. Thus, further multicenter studies focusing on anticoagulation in TIPS implantation are necessary to compare the individual regimens better.

Given all these limitations, this study indicates that a two-day heparinization can avoid early TIPS thrombosis; this thesis needs to be verified with a prospective study. Interventional radiologists should coordinate the anticoagulation regime in direct communication with the referring clinics, especially if the patient's coagulation status is unstable and additional anticoagulation therapy would significantly increase the risk of bleeding, for example, in cases involving esophageal varices.

## Material and methods

### Patients

In this retrospective study, all consecutive TIPS placements performed in a 4-year period (2014–2018) were examined. The local institutional ethics committee of the University Hospital Regensburg approved this retrospective analysis, and the study was performed following the relevant guidelines and regulations. The ethics committee waived informed consent (Ethics Committee, University of Regensburg, 93,040 Regensburg, Germany).

**Figure 5:**
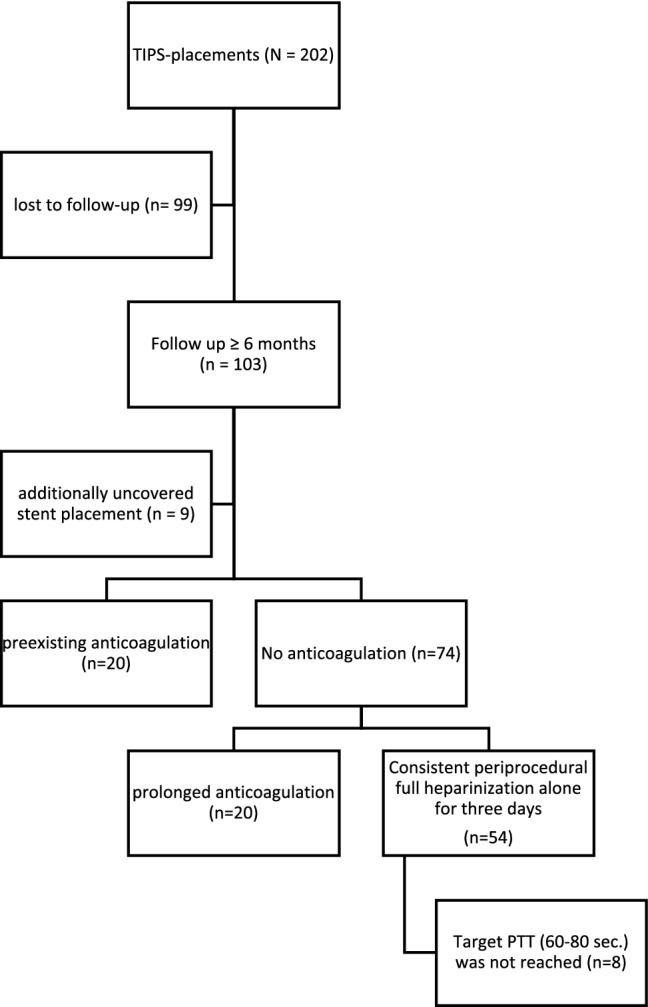
Flow-shard of inclusion and exclusion criteria. Search query revealed 202 new TIPS placements between 2014 and 2018. Ninety-nine patients were excluded as no post-interventional information was available (lost to follow-up). 103 TIPS placements were included in this retrospective analysis. 9 patients were excluded because of additionally uncovered stent placement. 20 patients had a preexisting anticoagulant drug and in 20 patients, the ward administered prolonged anticoagulation after the intervention. 54 patients were treated with periprocedural heparinization only.

Using a search query in the local RIS and PACS system, the TIPS interventions were identified. The inclusion criteria were an uncomplicated successful insertion of a TIPS without primary complications and sufficient follow-up over a 6-month period. A total of 103 TIPS placements were included in this retrospective analysis (Fig. 5). Nine patients were excluded because of additionally uncovered stent placement, resulting in a study population of 94 patients who underwent TIPS placement using PTFE-coated stents. The patient files were evaluated regarding the prescribed anticoagulation, the flow rate of the TIPS tract using color-coded Doppler sonography (CCDS), and occlusion of the TIPS.

Twenty patients had a preexisting anticoagulant drug (due to their medical history), which was continued after TIPS implantation. Of the 74 patients with no preexisting anticoagulation, all received anticoagulation medication according to the institute's standards. In 20 patients, the ward administered prolonged anticoagulation after the third day (n = 14, fractionated heparin; n = 2, prolonged unfractionated heparin; n = 2, thrombocyte aggregation inhibitor; n = 1, direct Xa inhibitor; n = 1 thrombocyte aggregation inhibitor + fractionated heparin). Ultimately, only 54 patients treated with periprocedural heparinization were included for further statistical analysis.

### Intervention

During intervention preparation, all patients received a contrast-enhanced CT or MRI before TIPS placement as a standard of care to determine the anatomic location of hepatic veins and portal vein branches and exclude existing lesions in the stent canal. Within 48 h before elective TIPS implantation, a comprehensive laboratory status was obtained, and patients were screened for contraindications, for example, decompensated hepatic encephalopathy. A team of experienced interventional radiologists performed all TIPS procedures under intubation anesthesia on Artis Zee® Biplane systems (Siemens Healthcare, Erlangen, Germany). After creating sterile conditions and covering the patient in a standardized manner, the right jugular vein was punctured under ultrasound control (Logic E9®, General Electric, Boston, MA, USA) using a micropuncture set (Cook Medical, Bloomington, IN, USA). The inferior vena cava (IVC) was then probed using a 0.014" guidewire to verify the correct position and to introduce an exchange cannula. After changing to a 0.035" wire (e.g., Glidewire®, Terumo, Tokyo, Japan) and stepwise dilatation of the jugular puncture site, a 30 cm long 10F sheath (Rösch-Uschida Punction-Set, Cook Medical, Bloomington IN, USA) was introduced. Via this access, a 5F MPA catheter (Cook Medical) was used to probe a hepatic vein, and a 260 cm Amplatz Super stiff wire (Boston Scientific, Malborough, MA, USA) was used to guide the 10F sheath into the hepatic vein. An angle-stable stiffening 14G cannula with a length of 51.5 cm was placed in the hepatic vein, and with the help of the direction indicator, the portal vein was punctured. A 100 cm Glidecath® (Terumo, Tokyo, Japan) was adjusted, and the correct position was proven by digital subtraction angiography (DSA). After successfully inserting the Amplatz superstiff wire using the catheter, the stiffening cannula was removed, and a 5F vascular sizing pigtail catheter (Merit Medical, South Jordan UT, USA) was inserted. The TIPS tract was visualized, and the pressure gradient of the portal system was measured using anesthesia monitors from Phillips and a pressure adapter (Edwards Lifescience, Irvine, CA, USA). The liver parenchyma was predilated with a 7–8 mm balloon, and the sheath was placed in the portal vein over the balloon. After the administration of 5,000 IU heparin, TIPS was inserted via the sheath. The stent was released via a retraction mechanism and dilated with a suitable balloon until the desired portosystemic pressure gradient (< 12 mmHg) was reached.

This study used the Gore® Viatorr® TIPS endoprosthesis in all interventions; it consists of a self-expanding nitinol (nickel-titanium) stent. The portal part of the stent prosthesis was uncovered over a distance of 2 cm, and a variable length (4–8 cm) was coated with expanded polytetrafluoroethylene (ePTFE). This coated part was placed intrahepatically, while the uncovered part remained in the portal vein. The diameter of these TIPSs ranged between 8 and 10 mm (mean 9.9 mm), with a primary usage of a diameter of 10 mm (n = 89).

### Postinterventional heparinization

Heparinization for two days post-interventionally with a target PTT of 60–80 s was performed in all TIPS placements. The PTT value before TIPS implantation and the maximum PTT in the postinventional course were noted to evaluate the degree of heparinization.

### Ultrasound

At our center, CCDS is performed as a standardized control examination within the first postinterventional two weeks and in the subsequent course at intervals of approximately three months. The flow values in the stent's proximal, middle, and distal third were analyzed. The definition for stent dysfunction was a flow velocity below 50 cm/s, indicating stent dysfunction^50,51^ or above 200 cm/s, indicating in-stent stenosis^24,52,53^.

### Characterization of TIPS dysfunction

There are no uniform criteria for TIPS dysfunction in the literature. Indicators include technical parameters such as flow velocity and portosystemic gradient (PSG) and clinical signs such as ascites and variceal bleeding. CCDS, along with invasive measurement of PSG, is considered the most appropriate noninvasive method for detecting TIPS dysfunction during follow-up and when clinical signs of dysfunction occur^52,54^. Hemodynamically significant stenoses are associated with a reduction in blood flow so that a reduction in flow velocity can be observed before and after the stenosed area of the stent. Flow velocities below 50–60 cm/s^50,51^ or below 80–90 cm/s^52^ can therefore be assumed to be caused by stent dysfunction. In the stenotic area itself, blood flow is faster due to the smaller lumen, and therefore, dysfunction is also assumed at flow velocities above 180–220 cm/s^24,52,53^. Rossle^55^ defined the corridor of a normal TIPS flow profile as between 40 cm/s and 200 cm/s. In sum, flow rates of ≤ 40 cm/s and ≥ 200 cm/s are considered pathological^55^.

In this study, TIPS dysfunction was defined as clinically relevantly impaired blood flow within the TIPS. TIPS stenosis was considered when the stent lumen was reduced to more than 50%, detected by ultrasound of the original diameter; an increase in PSG to > 12 mmHg was also interpreted as TIPS dysfunction. Stent occlusion was assumed when no flow was detected in the lumen or at the stent's proximal end. The primary patency rates and the primary assisted (after the first reintervention) patency rates are reported.

### Statistical analysis

All statistical analyses were done with IBM SPSS Statistics (version 27, Chicago, IL). Data are expressed as mean ± standard deviation (SD), and we used the non-parametric Mann–Whitney-Test and the Log-rang (Mantel-Cox) test to compare groups. All tests were two-sided, and values of p < 0.05 indicated a significant difference.

## Data Availability

The data that support the findings of this study are available within the article.
